# Analysis of the association between host genetics, smoking, and sputum microbiota in healthy humans

**DOI:** 10.1038/srep23745

**Published:** 2016-03-31

**Authors:** Mi Young Lim, Hyo Shin Yoon, Mina Rho, Joohon Sung, Yun-Mi Song, Kayoung Lee, GwangPyo Ko

**Affiliations:** 1 Department of Environmental Health Sciences, Graduate School of Public Health, Seoul National University, Seoul, Republic of Korea; 2 Division of Computer Science and Engineering, College of Engineering, Hanyang University, Seoul, Republic of Korea; 3 Department of Epidemiology, Graduate School of Public Health, Seoul National University, Seoul, Republic of Korea; 4 Department of Family Medicine, Samsung Medical Center, Sungkyunkwan University School of Medicine, Seoul, Republic of Korea; 5 Department of Family Medicine, Busan Paik Hospital, Inje University College of Medicine, Busan, Republic of Korea; 6 Center for Human and Environmental Microbiome, Seoul National University, Seoul, Republic of Korea; 7 N-Bio, Seoul National University, Seoul, Republic of Korea

## Abstract

Recent studies showing clear differences in the airway microbiota between healthy and diseased individuals shed light on the importance of the airway microbiota in health. Here, we report the associations of host genetics and lifestyles such as smoking, alcohol consumption, and physical activity with the composition of the sputum microbiota using 16S rRNA gene sequence data generated from 257 sputum samples of Korean twin-family cohort. By estimating the heritability of each microbial taxon, we found that several taxa, including *Providencia* and *Bacteroides*, were significantly influenced by host genetic factors. Smoking had the strongest effect on the overall microbial community structure among the tested lifestyle factors. The abundances of *Veillonella* and *Megasphaera* were higher in current-smokers, and increased with the pack-year value and the Fagerstrom Test of Nicotine Dependence (FTND) score. In contrast, *Haemophilus* decreased with the pack-year of smoking and the FTND score. Co-occurrence network analysis showed that the taxa were clustered according to the direction of associations with smoking, and that the taxa influenced by host genetics were found together. These results demonstrate that the relationships among sputum microbial taxa are closely associated with not only smoking but also host genetics.

Until recently, due to limitations in analytical methods, the airways of healthy humans were thought to be sterile^1^. However, recent studies using high-throughput next-generation sequencing (NGS) techniques have reported that the airways are not sterile (even in a healthy host) and harbor diverse microbial communities^2^,^3^. Several investigations have shown that changes in the composition of the airway microbiota are associated with the development of chronic lung diseases such as asthma, chronic obstructive pulmonary disease (COPD), and cystic fibrosis (CF)^3^,^4^,^5^. In addition, a decreased microbiota diversity has been reported in these diseases^4^,^6^. Therefore, the airway microbiota is considered to play an important role in host health and diseases, although the underlying mechanisms remain unclear. In this regard, it is important to characterize the “normal” airway microbiota in a large, healthy population in order to gain insights into the basic characteristics of airway microbiota composition, to identify possible causes of airway microbiota variations across healthy individuals, and to use the information as a reference for identifying specific microbial signatures of microbial dysbiosis-related lung diseases.

Few studies to date have examined associations between host genetic or lifestyle factors and the airway microbiota. For example, it has been reported that the use of antibiotics, a conventional approach to control chronic respiratory infections in CF patients, is related to the decreased richness of the sputum microbiota^7^,^8^. Although smoking is another environmental factor that can lead to alterations in the composition of the airway microbiota by directly affecting the airway environment^9^, at this time few studies have assessed the links between smoking and the airway microbiota^4^,^10^,^11^,^12^, and little is known regarding how smoking affects a normal balanced microbiota. One of these previous studies compared the bronchoalveolar lavage (BAL) communities of healthy non-smokers (n = 45) and smokers (n = 19), but did not observe significant differences in the microbial composition^10^. These findings should be confirmed in a larger study. In terms of host genetic effects, host genetics play a role in shaping the gut microbiota^13^. However, whether the airway microbiota of healthy individuals is influenced by host genetic factors has yet to be determined. In this context, our knowledge of the airway microbiota and its relationship with various host-associated factors remains limited.

In this study, we comprehensively investigated the associations of sputum microbial composition with host factors using a total of 257 sputum samples (as a proxy for lower respiratory tract microbial composition) and their metadata from healthy Korean monozygotic (MZ) twins, dizygotic (DZ) twins, and their families in order to characterize the effects of host genetics and lifestyle factors such as smoking, alcohol consumption, and physical activity on the sputum microbiota.

## Results

### Participant characteristics

A total of 257 sputum samples from individuals enrolled in the Healthy Korean Twin Study cohort were collected as described in a previous study^14^. This study population comprised 74 MZ twin pairs (n = 148) and their parents or siblings (n = 58), as well as 14 DZ twin pairs (n = 28) and their parents or siblings (n = 23). The average age of the subjects was 46.15 (±12.70) years, and 57.98% were female (Table 1). All subjects had not taken antibiotics within three months prior to sampling. The participants had no current clear symptoms of chronic lung diseases, such as asthma, and no history of diagnosed asthma and COPD. Demographic and lifestyle characteristics of the study participants are shown in Table 1.

### Core sputum microbiota of a healthy population

**Figure 1:**
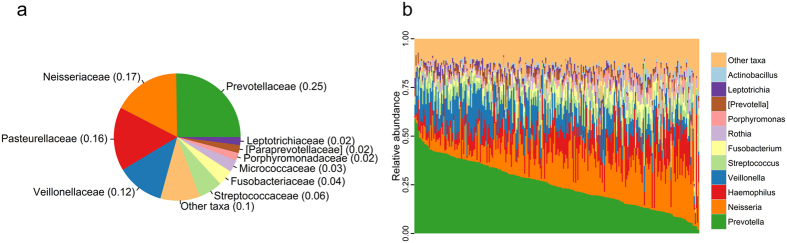
Composition of the sputum microbiota. (**a**) Pie chart of the average relative abundances of the families. (**b**) Bar chart of relative abundances of the genera across each sampled sputum microbiome.

A mean of 46,311 sequences (±30,529 SD) per sample were obtained by 16S rRNA gene sequencing from sputum samples collected from each subject. Proteobacteria (34%), Bacteroidetes (32%), Firmicutes (21%), Fusobacteria (6%), and Actinobacteria (4%) constituted the five most abundant bacterial phyla. At the family level, Prevotellaceae (25%), Neisseriaceae (17%), Pasteurellaceae (16%), and Veillonellaceae (12%) were the most abundant families (Fig. 1a). These families were present in all samples, and constituted the family-level core sputum microbiota. At the genus level, *Prevotella* (25%) and *Neisseria* (16%), followed by *Haemophilus* (11%), *Veillonella* (11%), and *Streptococcus* (6%) were most abundant in this healthy population, and were present as members of the genus-level core microbiota (Fig. 1b). Notably, *Prevotella* showed substantial inter-individual variation. All members of the core sputum microbiota (>1% average relative abundance and 100% prevalence) at the family and genus levels are represented in Fig. 1.

### Host genetic effects on the sputum microbiota

**Figure 2:**
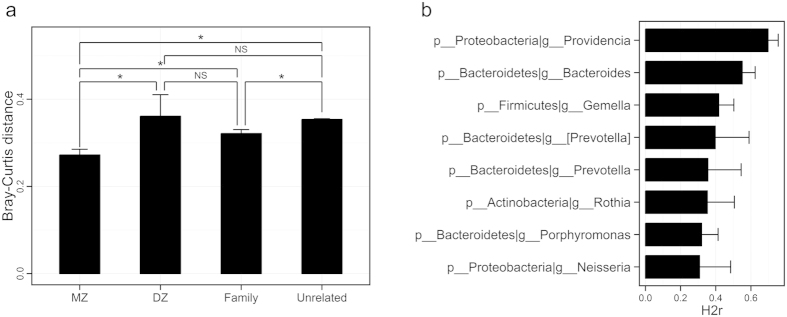
Influences of host genetic factors on the sputum microbiota. (**a**) Bray-Curtis distances between the sputum microbiota of monozygotic (MZ) twin pairs, dizygotic (DZ) twin pairs, family members of the twin pairs, and unrelated individuals belonging to the various twin families (mean ± s.e.m.; *P < 0.05 for two sample t-test via 1,000 Monte Carlo permutations). (**b**) Heritability estimates (H2r) of sputum microbial taxa (mean ± s.e.m.). Genera with significant heritability (H2r > 0.3) are shown.

We investigated whether host genetics influence sputum microbiota. By comparing Bray-Curtis distances between MZ twin pairs, DZ twin pairs, family members of the twin pairs, and unrelated individuals belonging to the various twin families, we found that the sputum microbiota of MZ twin pairs were significantly more similar to one another than were those of DZ twin pairs (p-value = 0.046, two sample t-test via 1,000 Monte Carlo permutations; Fig. 2a). As expected, MZ twin pairs had more-similar microbial community structures than family members and unrelated individuals (p-value = 0.004 and p-value < 2.2e-16; Fig. 2a). This result suggested that the overall community structure of sputum microbiota could be partially modulated by host genetics.

Before identifying specific microbial taxa with heritability using Sequential Oligogenic Linkage Analysis Routines (SOLAR)^15^, we first estimated the heritability (H2r) of smoking after adjusting for age and sex. We found that the pack-year smoking history was significantly heritable (H2r = 0.462, p = 0.002), while the Fagerstrom Test of Nicotine Dependence (FTND) was not (H2r = 0.141, p = 0.192). Next, we calculated the heritability estimate of each taxon after adjusting for pack-years of smoking as well as age and sex in order to assess genetic influence on the abundance of each taxon, regardless of pack-years of smoking. Among the 71 tested taxa with >0.1% average relative abundance, 39 taxa (54.9%) were significantly heritable. The heritability estimates of these taxa ranged from 0.19 to 0.70 (Supplementary Table S1). At the genus level, we observed significant moderate (H2r = 0.3~0.6) to high (H2r > 0.6) heritability for *Providencia* (H2r ± SE; 0.70 ± 0.06), *Bacteroides* (0.55 ± 0.07), *Gemella* (0.42 ± 0.09), *Prevotella* (0.36 ± 0.19), *Rothia* (0.35 ± 0.15), *Porphyromonas* (0.32 ± 0.09), and *Neisseria* (0.31 ± 0.18) (Fig. 2b). Collectively, these data demonstrate that host genetic factors could specifically affect a portion of the sputum microbiota.

### Relationship between smoking and sputum microbiota

**Figure 3:**
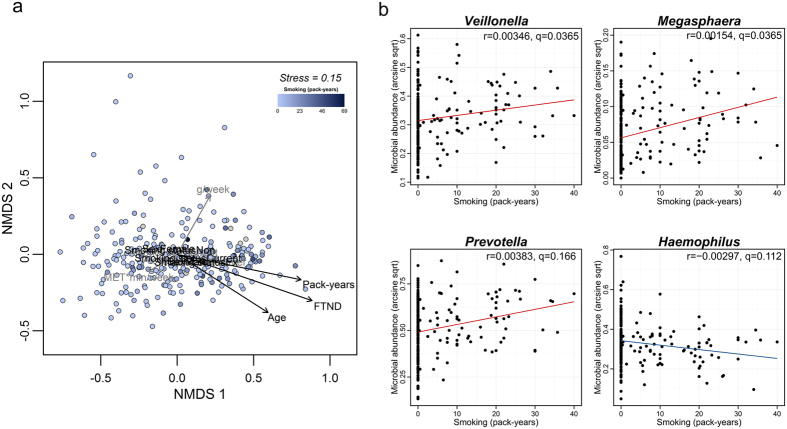
Associations of lifestyle factors with sputum microbiota. (**a**) Non-metric multidimensional scaling (NMDS) plot of genus composition. Points represent samples that are colored according to pack-years of smoking. Gray points indicate samples with missing pack-year values. The black arrows indicate significant correlations with the ordination, whereas the gray arrows indicate non-significant correlations. (**b**) Significant associations between pack-years of smoking and microbial taxa. The r-coefficient and q-value shown in each plot were determined by MaAsLin analysis.

We first investigated the relationship between the overall sputum microbial community structure and lifestyle factors (smoking history (pack-years), alcohol drinking (g/week), and physical activity (MET∙min/week)) by conducting non-metric multidimensional scaling (NMDS). We found that pack-years of smoking was significantly correlated with the NMDS ordination of the microbial community structure (envfit; R^2^ = 0.061, p-value = 0.012); increasing pack-year values was represented by a vector pointing to the right of the NMDS plot (Fig. 3a). The remaining lifestyle factors were not significantly correlated with the NMDS ordination of the microbial community structure (envfit; alcohol consumption: R^2^ = 0.017, p-value = 0.147; physical activity: R^2^ = 0.004, p-value = 0.645). Additionally, age and sex showed significant correlations with the NMDS ordination (envfit; age: R^2^ = 0.044, p-value = 0.012; sex: R^2^ = 0.032 p-value = 0.004). The smoking status (non-smoker, ex-smoker, and current-smoker) and the FTND score were also significantly correlated with the NMDS ordination (envfit; smoking status: R^2^ = 0.030, p-value = 0.016; FTND: R^2^ = 0.077, p-value = 0.002). Therefore, our data showed that smoking strongly influenced the structure of the sputum microbial communities.

To identify specific microbial taxa significantly associated with lifestyle factors while accounting for age, sex, and family structures, we performed a multivariate association with linear models (MaAsLin) analysis^16^. Among the lifestyle factors, the variable of pack-year smoking history showed the highest number of significant associations with microbial taxa (n = 17): pack-year of smoking was positively associated with taxa from the Firmicutes phylum, including the *Veillonella* and *Megasphaera* genera, and the *Prevotella* genus, while this variable was inversely associated with taxa from the Proteobacteria phylum, including the *Eikenella* and *Haemophilus* genera (Fig. 3b and Supplementary Table S2). In addition, we observed significant associations of specific microbial taxa with alcohol consumption (n = 2) and physical activity (n = 1). For example, alcohol consumption was positively associated with taxa from Actinobacteria *Actinomyces*, and physical activity was negatively associated with the Firmicutes Lachnospiraceae, respectively (Supplementary Table S2). When testing for associations of the microbial taxa with the smoking status, instead of a pack-year variable, we found that the genera *Veillonella* and *Megasphaera* were significantly enriched in current-smokers compared to non-smokers (Supplementary Figure S1a and Supplementary Table S3). Consistent with these results, higher FTND scores were also associated with increased abundances of the *Veillonella* and *Megasphaera* genera. The *Haemophilus* genus was negatively associated with FTND score (Supplementary Figure S1b Supplementary Table S4). These results suggested that smoking is a major environmental factor that not only drives the difference in microbial community structure between samples but also affects the abundance of specific microbial taxa.

We additionally explored the association between lifestyle variables and alpha diversity indexes (Chao1 richness and Shannon diversity index). There were no significant correlations between the lifestyle factors and alpha diversity (Spearman’s correlation; ρ = 0.03~0.12, p-value > 0.05, Supplementary Figure S2). In agreement with this, both richness and diversity estimates were not significantly different regarding age or sex (Spearman’s correlation; ρ = 0.06, p-value > 0.05, Wilcoxon rank sum test; p-value > 0.05, Supplementary Figure S2).

### The influence of smoking and host genetics on specific taxa of the sputum microbiota

**Figure 4:**
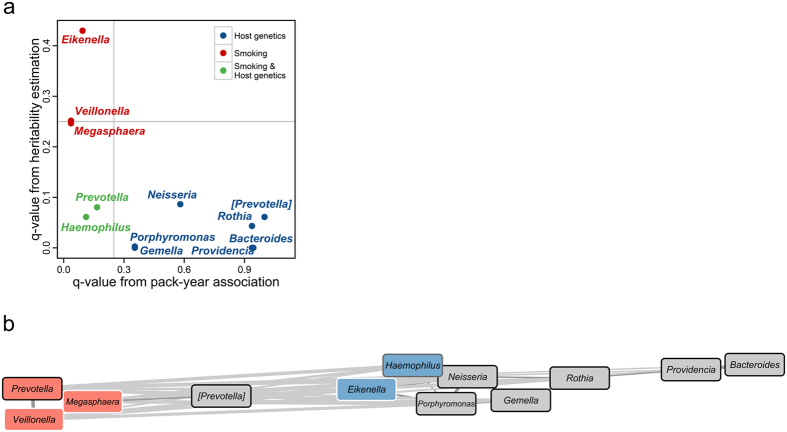
Effects of smoking and host genetics on specific microbial taxa. (**a**) Scatter plot of q-values for the association of each genus with pack-years of smoking (x-axis; from MaAsLin analysis) versus q-values for the heritability of each genus (y-axis; from SOLAR analysis). Red, blue, and green points represent genera that are significantly associated with pack-years of smoking, host genetics, and both, respectively. (**b**) Correlation network of the smoking (pack-years)- or host genetics-associated genera. Pink and light blue nodes indicate genera positively and negatively associated with smoking, respectively. Gray nodes indicate genera not significantly associated with smoking. The border of each node is colored according to the heritability (black: significant heritability with H2r > 0.3; gray: significant heritability with H2r < 0.3; white: non-significant heritability). Edges represent significant positive (dark gray) or negative (light gray) correlations between the nodes they connect. The width of the edge represents the degree of correlations.

Our findings indicate that both host genetics and smoking influence specific taxa of the sputum microbiota. To increase our understanding of the interactions among sputum microbial communities, smoking, and host genetics, we performed a further analysis by combining the results of the following two analyses: SOLAR analysis (estimating the heritability of specific taxa; Supplementary Table S1) and MaAsLin analysis (testing for the association between smoking and specific taxa; Supplementary Table S2). For the genera shown to be significantly associated with pack-year of smoking (hereafter, “smoking-associated genera”) or the genera with significant moderate to high heritability (hereafter, “host genetics-associated genera”), the q-values from MaAsLin (x-axis) were plotted as individual points against the corresponding q-values from SOLAR (y-axis) in Fig. 4a. In most cases, the genera showed significant associations with either smoking or host genetics. *Haemophilus* and *Prevotella* was the only genera found to be significantly associated with both factors.

To reveal relationships among the smoking-associated genera and the host genetic-associated genera, we performed co-occurrence analysis based on Spearman’s rank correlations. There were 39 significant correlations (Fig. 4b). We observed that the smoking-associated genera co-occurred according to the tendency toward smoking. For example, *Veillonella,* which was positively associated with pack-year values, had positive correlations with *Megasphaera* and *Prevotella* (Spearman’s correlation; ρ = 0.64 and 0.54, respectively). Other smoking-associated genera, such as *Haemophilus* and *Eikenella*, which were negatively associated with pack-year values, co-occurred together (Spearman’s correlation; ρ = 0.27). On the other hand, *Veillonella* was anti-correlated with *Haemophilus* and *Eikenella* (Spearman’s correlation; ρ = −0.23 and −0.28, respectively). Additionally, we observed that the genera associated only with host genetics were closely linked, forming a *Rothia*-centered cluster. A strong positive correlation was observed between the two most-heritable genera, *Providencia* and *Bacteroides* (Spearman’s correlation; ρ = 0.66), while the former was also positively correlated with *Rothia* (Fig. 4b). The data demonstrated that relationships among members of the sputum microbiota are associated with both smoking and host genetics.

## Discussion

In this study, we show that both host genetics and smoking influence the abundance of specific taxa of the sputum microbiota in healthy adult populations. This finding is important because identification of the host factors that have influence on the sputum microbiota and the specific taxa that is associated with those host factors provides a crucial information for prevention of microbial dysbiosis-related lung diseases.

Most previous studies on the airway microbiota have focused on identifying the differences in the microbial composition of patients with lung diseases and healthy controls. By contrast, in this study, we explored the characteristics of a normal sputum microbiota in healthy populations. We employed sputum samples as a proxy for lower respiratory tract microbial composition. So far, sputum would be the most suitable sample for analyzing the airway microbiota of a large, healthy population because sputum samples can be obtained less invasively and more readily. Our relatively large sample size (n = 257) allowed us to determine the core sputum microbiota and to investigate associations of various lifestyle factors with the sputum microbiota with high precision. Furthermore, our study population, consisting of twins and their families, allowed us to estimate the heritability of each member of the microbiota. Therefore, we detected independent effects of lifestyle factors on the sputum microbiota taking into consideration host genetic factors in this study.

We characterized the composition of the normal sputum microbiota and identified the core members, including *Prevotella*, *Neisseria*, *Veillonella*, and *Streptococcus* (Fig. 1). Our data are consistent with previous studies using sputum samples from a small number of healthy subjects^17^,^18^,^19^. Significant portion of the sputum microbiota overlapped with commonly observed taxa of the oral microbiota^20^,^21^. This is not surprising because the oral cavity and the respiratory tract are anatomically contiguous. There is some chance of oral microbiota contamination in the sputum samples. However, previous studies demonstrated significant differences in abundances of specific bacteria between oral washes and BAL samples of healthy subjects^10^ and distinct microbial profiles of oral washes and sputum samples from CF patients^22^. Based on our principal coordinate analysis and correlation analysis of oral and sputum microbiota in our study subjects (unpublished data), sputum microbiota was clearly distinctive from oral microbiota. Previous studies and our data suggest that observation of the oral microbes in the airway samples is not the result of simple oral contamination, but may indicate actual physiological condition. In addition, we washed the mouth of the subjects prior to obtaining sputum samples in order to minimize oral contamination in our study^23^,^24^,^25^. Therefore, while contamination with oral microbes is possible, the degree of any such contamination and its effect on the analysis thereof should be very minimal.

Host genetics was an important factor influencing the composition of the sputum microbiota. The overall microbiota structures from MZ twin pairs were significantly more similar compared to those from DZ twin pairs, family members, or unrelated individuals. These results suggest that the sputum microbiota are at least in part genetically influenced (Fig. 2a). As shown in Fig. 2b, many specific sputum microbial taxa showed significant heritability estimates. When we estimated the heritability of specific microbes, we adjusted for age, sex, and smoking history. Therefore, the high heritability of some members of the sputum microbiota remains valid regardless of these factors. Among them, *Providencia* (belonging to Proteobacteria) was the most highly heritable taxon (Fig. 2b). Five species of *Providencia* have been isolated from various human specimens, such as urine, sputum, and stool^26^. Although *P. stuartii* has long been recognized as an opportunistic pathogen responsible for urinary tract infections in long-term care patients with chronic indwelling urinary catheters^27^, the effect of *Providencia* on the human airway remains largely unknown. Therefore, how *Providencia* is influenced by host genetics and its biological effect on the airway should be further investigated. Among the other sputum microbiota taxa, *Rothia* exhibited significant heritability. This finding is interesting because this taxon was also identified in the gut and was found to be highly heritable in the gut microbiome data set of American twins^13^. Therefore, *Rothia* may be specifically influenced by host genetic factors regardless of the body site in which they reside. There may be common phenotypic factors between bronchial and intestinal epithelial cells. Further studies are required to determine whether the heritability of this taxon can also be observed at other body sites and to identify the host alleles that contribute to the heritability. Hampton *et al.* reported that the sputum microbiota of 13 pediatric CF patients was susceptible to environmental influences rather than host genetic effects^28^. This study suggests that both lifestyle and disease status can affect the composition of the sputum microbiota.

Among the lifestyle factors investigated, smoking had the strongest effect on the overall microbial community structure (Fig. 3a). This result is reasonable because cigarette smoke contains a wide range of components—including carcinogens, toxins, and oxidants^29^—which can alter the airway environment by inducing cellular damage and inflammation^30^. We further identified a specific set of microbial taxa significantly associated with smoking. Interestingly, we observed that the pack-year value was positively associated with abundance of the *Veillonella* and *Megasphaera* genera and all taxa to which these genera belong (Firmicutes; Clostridia; Clostridiales; Veillonellaceae) (Supplementary Table S2). Changes in the abundances of the *Veillonella* genus were similar to those reported in the study by Charlson and colleagues^12^ that compared the oropharynx of 29 cigarette smokers and 33 non-smokers, and were confirmed by multivariate analysis using the smoking status or the FTND score in this study. The *Veillonella* genus is part of the normal flora of the oral, respiratory, and intestinal tracts of humans. A positive correlation of the relative abundance of *Veillonella* in the BAL microbiota with exhaled nitric oxide (a non-invasive measure of airway inflammation) in asymptomatic individuals has been reported^11^. *Veillonella* is a Gram-negative bacterium that can induce an inflammatory host response by producing lipopolysaccharides (LPS)^31^. We also found that *Haemophilus* and *Eikenella* were present at greater abundance in individuals with lower pack-year values (Fig. 3b and Supplementary Table S2). This result is consistent with a previous report showing that abundance of *Haemophilus* in the oropharynx was higher in nonsmokers compared to smokers^12^. Interestingly, non-typeable *H. influenza* is a well-known pathogen that causes respiratory tract infections in COPD patients^32^, but the abundance of *Haemophilus* decreased with increasing pack-year values in the present study. Since members of the *Haemophilus* genus are common resident bacteria of the human airways^33^, further studies are required to explore the effect of smoking on specific species or strains of this genus. These observations suggest that smoking alters the abundance of specific members of the sputum microbiota, such as *Veillonella*, which can lead to an inflammatory condition or an environment in which pathogenic bacteria can thrive, and subsequently contribute to the development of smoking-related lung diseases.

In the correlation network, we observed a significant negative relationship between the two smoking-associated taxa, *Haemophilus* (Proteobacteria) and *Veillonella* (Firmicutes) (Fig. 4b). Since we used Spearman’s correlation measures for the relative abundances of the taxa, a high abundance of one member of the microbiota can be accompanied by a reduction in the relative abundances of other members. However, this co-exclusion relationship suggests that these taxa require different environmental conditions to survive. Indeed, *Haemophilus* is a Gram-negative, aerobic/facultative anaerobic bacterium belonging to the Pasteurellaceae family^34^, whereas *Veillonella* is a genus of anaerobic Gram-negative cocci^35^. Therefore, changes in oxygen levels of the airway environment caused directly by smoking or indirectly by smoking-related host responses may play a role in their co-exclusion relationships, although details on the mechanisms are not yet available. In addition, the most heritable genus, *Providencia*, was present together with the second-most heritable genus, *Bacteroides*, in the co-occurrence network (Fig. 4b). These taxa were present at low relative abundances (0.11–0.47%) in the sputum microbiota, and each belongs to one of two distinct phyla. Thus, our result suggests that these minor, heritable taxa may interact in a cooperative manner, and have a preference for a similar airway environment that is under the influence of host genetics. By network analysis, we showed that the relationships among microbial taxa are closely associated with not only smoking but also host genetics.

In summary, the data presented here indicate that both host genetics and lifestyle contribute to determining the composition of the normal sputum microbiota of healthy adults. Further studies using longitudinal sample sets are required to understand the causative associations of smoking with specific taxa and to identify the health consequences of smoking-related changes in the sputum microbiota. Our findings linking host genetics, lifestyle, and their sputum microbiota can be used to establish future therapeutic biological markers for microbiome-mediated lung diseases.

## Methods

### Study subjects and sputum sample collection

The sputum samples and associated metadata were obtained from participants enrolled in the Healthy Twin Study in Korea^36^. Samples were excluded from this study if the participants had received antibiotic treatment or cold medication within the past three months. In addition, samples from participants with current clear symptoms of lung disease, such as asthma, or with a history of diagnosed asthma and COPD were excluded. The final study subjects consisted of 257 individuals, including 74 pairs of MZ twins (n = 148), 14 pairs of DZ twins (n = 28), and their parents or siblings (n = 81).

Prior to sputum induction, distilled water was used as a mouth rinse to minimize contamination of sputum samples with oral microbes^23^,^24^,^25^. Then with five deep breaths to allow opening of the airways, sputum samples were induced by coughing forcefully several times. Each serially induced sputum sample was <1 ml (700–800 μl) in volume and collected in sterile plastic tubes. Samples were stored at −70 °C until use. All subjects were asked to complete a questionnaire about smoking, alcohol consumption, and physical activity. The pack-year of smoking was calculated as packs per day multiplied by years of smoking. Weekly consumption of alcohol (g/week) was calculated as the frequency of drinking per week multiplied by the mean amount of alcohol consumption at each drinking session. Physical activity was assessed using the Korean version of the International Physical Activity Questionnaire^37^. Nicotine dependence was measured using the FTND^38^. The study was approved by the Institutional Review Board of Samsung Medical Center (IRB file No. 2005-08-113), Busan Paik Hospital (IRB file No. 05-037), and Seoul National University (IRB file No. 144-2011-07-11). All experiments were performed in accordance with relevant guidelines and regulations. Informed consent was obtained from all participants.

### DNA extraction and 16S rRNA gene sequencing

Sputum specimens were carefully transferred into sterile 1.5ml tubes, then mechanically homogenized by vortexing at maximum speed with MoBio vortex adapter for 10 min. For samples that were not thoroughly homogenized within 10 min, we extended the vortexing time to 20 min. The samples were then washed twice with cold phosphate-buffered saline (PBS), followed by DNA extraction using PowerSoil DNA Isolation Kit (MO BIO Laboratories, Carlsbad, CA, USA) according to the manufacturer’s instructions. Samples were additionally homogenized with bead-beating procedure included in the aforementioned kit. DNA was eluted in 50 μL of MoBio elution buffer and stored at −20 °C until use.

The 16S rRNA genes were amplified using the 515F and 806R primers for the V4 region, as described previously^39^. PCR products were purified using MO BIO UltraClean PCR Clean-Up Kit (MO BIO Laboratories, Carlsbad, CA, USA), and subsequently quantified using the KAPA Library Quantification Kit (KAPA Biosystems, Woburn, MA, USA). Sequencing was performed on the MiSeq platform using a paired-end 2 × 300-bp reagent kit according to the manufacturer’s instructions (Illumina, San Diego, CA, USA).

### Sequence analysis

Sequences generated from the MiSeq run were processed using QIIME v1.8.0^40^ and were clustered into 97% identity using a two-step open-reference operational taxonomic unit (OTU) picking method. Taxonomy was assigned to each OTU using the Ribosomal Database Project (RDP) classifier based on the 13_5 revision of the Greengenes database. Read counts from the OTU table were collapsed at six levels from domain to genus, and subsequently converted to relative abundances for further analysis. Unclassified taxa at a given taxonomic level were excluded from the analysis.

### Bioinformatics analysis and statistical tests

The Bray-Curtis distances between each sample were calculated using the genus-level abundance profiles to evaluate the similarity between microbial communities from MZ twin pairs, DZ twin pairs, family members of the twin pairs, and unrelated individuals belonging to the various twin families. The statistical significance of differences was evaluated using a two-sample t-test via 1,000 Monte Carlo permutations. For the test, 20% of the unrelated pairs selected randomly were used to reduce the number of unrelated pairs.

Heritability for each microbial taxon was estimated using a variance component method implemented in SOLAR^15^. Low-abundance taxa (average relative abundance <0.1%) were excluded from this analysis in order to minimize the effect of the potential background signals and to meet the analytic conditions of SOLAR. The data of microbial abundances from all MZ/DZ twin pairs and their parents and siblings was included into this analysis. The microbial abundances were used as quantitative phenotypes. Inverse normal transformation was performed for the microbial abundances with the function “inorm” implemented in SOLAR prior to variance decomposition analysis. Total phenotypic variance (σp^2^) in microbial abundances after consideration of covariates (age, sex, and pack-years of smoking) was partitioned into additive genetic component (σa^2^), a shared environmental component within a family (σc^2^), and an individual specific unique environmental component (σe^2^). Heritability estimates (H2r) were calculated as the proportion of the total phenotypic variance explained by additive genetic effects (σa^2^/σp^2^). Significant heritability was considered when an FDR-corrected p-value (q-value) was <0.25 and a p-value was <0.05.

NMDS analysis was performed using MetaMDS function within the vegan package in R^41^ based on the Bray–Curtis distance measure obtained for the genus-level data. A total of 20 iterations were performed and the NMDS dimension with the lowest stress was retained for data visualization. Vectors of metadata were fitted onto the NMDS ordination plot using the envfit function of the vegan package. This function calculates the goodness-of-fit values (R^2^) for metadata onto the NMDS ordination plot and their significances using 999 random permutations.

Multivariate analysis was conducted using MaAsLin^16^ to test for associations of microbial abundances (at all taxonomic levels from domain to genus) with lifestyle factors, such as smoking (pack-years, smoking status, or FTND score), alcohol consumption, and physical activity. In this analysis, age and sex were used as fixed effects, and MZ twin ID and family ID were used as random effects. Only taxa with average abundances across all samples >0.1% were included in the MaAsLin analysis. Association was considered significant at q-value of below 0.25, a cut-off used in previous microbiome studies^16^,^42^,^43^,^44^.

Alpha diversity indexes—the Chao1 index (richness) and the Shannon index (diversity)—were calculated with rarefied data (10,000 sequences per sample) using QIIME^40^. Wilcoxon rank sum test and Spearman’s correlation test were used to evaluate the significance of the association between these index measures and host factors.

The co-occurrence relationships between the relative abundances of taxa (at the genus level) found to be significantly heritable or significantly associated with smoking were evaluated by calculating Spearman’s ranked correlation. Networks of co-occurring taxa were visualized using the edge-weighted spring embedded layout algorithm in Cytoscape^45^. In the network, each node represents a genus, and pairs of nodes were linked by edges (correlation coefficients) only when their q-values were <0.25 and p-values were <0.05.

## Additional Information

**Accession codes:** The sequences from this study were deposited in the European Nucleotide Archive under the study accession number ERP010734.

**How to cite this article**: Lim, M. Y. *et al.* Analysis of the association between host genetics, smoking, and sputum microbiota in healthy humans. *Sci. Rep.* **6**, 23745; doi: 10.1038/srep23745 (2016).

## Supplementary Material

Supplementary Information

## Figures and Tables

**Table 1:** Characteristics of participants in this study.

Variable	Overall(n = 257)	MZ twin		DZ twin	
		Twin pairs(n = 148)	Family members(n = 58)	Twin pairs(n = 28)	Family members(n = 23)
Females, n (%)	149 (57.98)	84 (56.76)	34 (58.62)	16 (57.14)	15 (65.22)
Age (years)	46.15 (12.70)	41.22 (8.67)	56.93 (14.27)	40.5 (7.31)	58.9 (11.58)
BMI (kg/m^2^)	23.63 (3.21)	23.33 (2.86)	23.95 (3.59)	23.19 (3.55)	24.7 (3.53)
Smoking (pack-years)^§^	5.12 (10.23)	5.09 (10.03)	6.66 (12.69)	3.16 (6.54)	3.82 (7.89)
Smoking status, n (%)*					
Non-smoker	170 (66.15)	92 (62.16)	41 (70.69)	19 (67.86)	18 (78.26)
Ex-smoker	33 (12.84)	19 (12.84)	8 (13.79)	4 (14.29)	2 (8.70)
Current-smoker	54 (21.01)	37 (25.00)	9 (15.52)	5 (17.86)	3 (13.04)
Alcohol consumption (g/week)^#^	101.13 (239.08)	104.98 (204.25)	95.8 (281.24)	80.89 (108.15)	83.40 (139.33)
Alcohol use, n (%)					
Non-use	38 (14.79)	13 (8.78)	14 (24.14)	4 (14.29)	7 (30.43)
Former use	31 (12.06)	19 (12.84)	10 (17.24)	0 (0)	2 (8.70)
Current use	188 (73.15)	116 (78.38)	34 (58.62)	24 (85.71)	14 (60.87)
Physical activity (MET∙min/week)^$^	5885.75 (9827.53)	5233.04 (9854.18)	6131.18 (8760.54)	6969.73 (12388.92)	8865.05 (12055.24)

Data are presented as means (standard deviation) unless otherwise stated.

*No significant difference in smoking prevalence between MZ and DZ twins was observed (p-value = 0.70, Fisher’s exact test).

Missing data (numbers for overall, MZ twin pairs, family members of MZ twins, DZ twin pairs, and family members of DZ twins, respectively).

^§^Six subjects with missing data (6, 4, 1, 1, 0);

^#^Twenty-six subjects with missing data (26, 14, 9, 0, 3);

^$^Thirty-seven subjects with missing data (37, 23, 7, 4, 3).
