# Supplementary material for: Analysis of the association between host genetics, smoking, and sputum microbiota in healthy humans
Source: Sci Rep. 2016 Mar 31;6:23745. doi: 10.1038/srep23745 (PMC4814871; doi:10.1038/srep23745)
Supplement: Supplementary Information [file srep23745-s1.pdf]

Supplementary information

Analysis of the association between host genetics, smoking, and sputum microbiota in healthy humans

Mi Young Lim, Hyo Shin Yoon, Mina Rho, Joohon Sung, Yun-Mi Song, Kayoung Lee, GwangPyo Ko

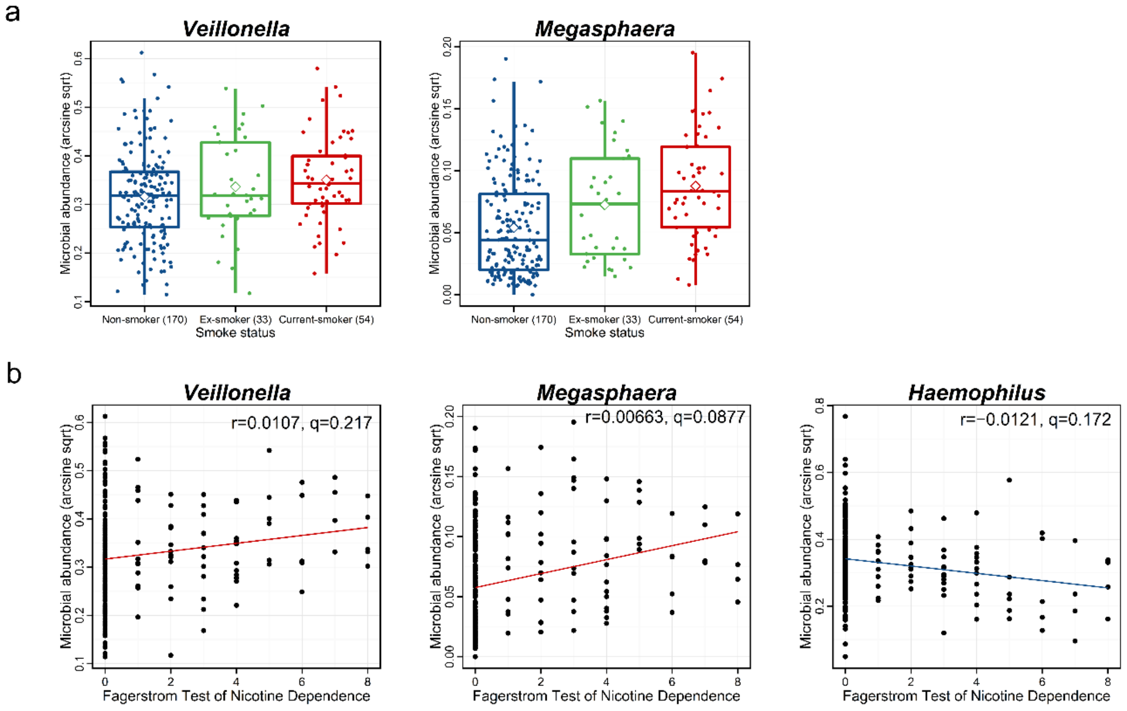

Supplementary Figure S1. Significant associations of microbial taxa with (a) smoking status and (b) Fagerstrom Test of Nicotine Dependence (FTND) scores. Significances were determined by MaAsLin analysis.

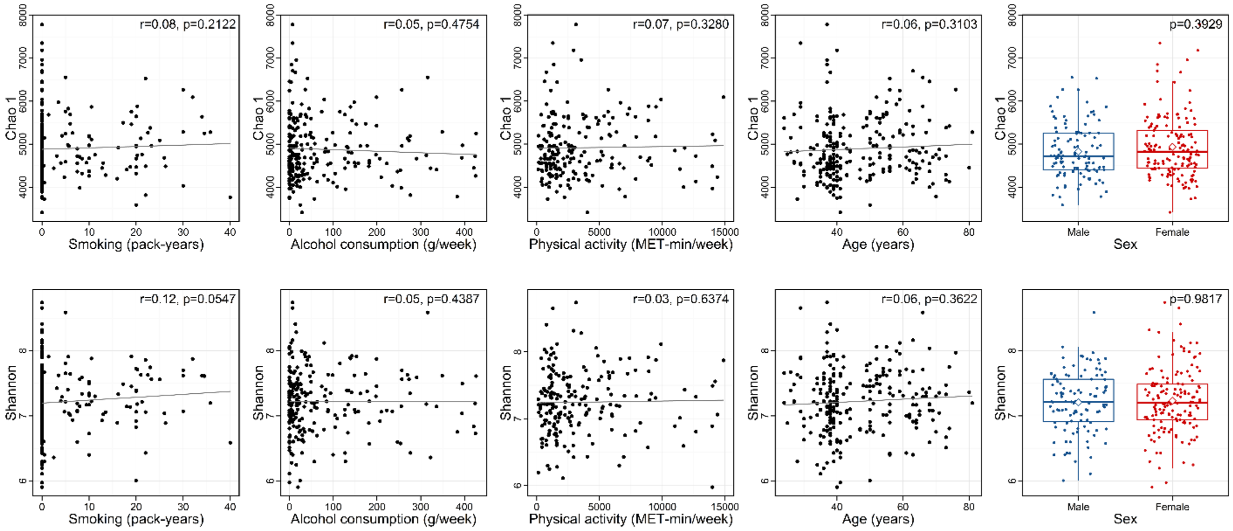

Supplementary Figure S2. Choa1 richness (upper panel) and Shannon diversity index (lower panel) according to lifestyle factors (smoking, alcohol consumption, and physical activity), age, and sex. The alpha diversity indexes were calculated from rarefied OTU table. The r-coefficient and p-value shown in each scatter plot were calculated by Spearman's rank correlation. The p-value shown in each box plot was calculated by Wilcoxon rank-sum test.

Supplementary Table S1. Heritability of the sputum microbiota.

Taxon	H2r	p.H2r	se.H2r	FDR. H2r	C2	p.C2	se.C2	FDR. C2	p.sex	p.age	p.PY
Bacteria	0.324	0.000	0.093	0.002	0.000	NA	NA	NA	0.806	0.511	0.677
Bacteria|Actinobacteria	0.310	0.029	0.159	0.063	0.116	0.185	0.134	0.400	0.265	0.097	0.411
Bacteria|Actinobacteria|Actinobacteria	0.343	0.017	0.156	0.049	0.105	0.204	0.132	0.400	0.244	0.111	0.498
Bacteria|Actinobacteria|Actinobacteria|Actinomycetales	0.345	0.017	0.154	0.049	0.099	0.214	0.129	0.400	0.254	0.109	0.481
Bacteria|Actinobacteria|...|Actinomycetales|Actinomycetaceae	0.266	0.002	0.091	0.009	0.000	NA	NA	NA	0.835	0.752	0.099
Bacteria|Actinobacteria|...|...|Actinomycetaceae|Actinomyces	0.262	0.002	0.092	0.010	0.000	NA	NA	NA	0.800	0.774	0.096
Bacteria|Actinobacteria|...|Actinomycetales|Micrococcaceae	0.352	0.013	0.153	0.043	0.145	0.126	0.133	0.400	0.242	0.102	0.811
Bacteria|Actinobacteria|...|...|Micrococcaceae|Rothia	0.352	0.013	0.153	0.043	0.145	0.126	0.133	0.400	0.244	0.102	0.821
Bacteria|Actinobacteria|Coriobacteriia|Coriobacteriales|Coriobacteriaceae	0.194	0.027	0.103	0.061	0.000	NA	NA	NA	0.142	0.063	0.061
Bacteria|Actinobacteria|...|...|Coriobacteriaceae|Atopobium	0.190	0.029	0.103	0.063	0.000	NA	NA	NA	0.138	0.060	0.060
Bacteria|Bacteroidetes	0.358	0.001	0.113	0.004	0.000	NA	NA	NA	0.087	0.065	0.029
Bacteria|Bacteroidetes|Bacteroidia|Bacteroidales	0.387	0.000	0.113	0.002	0.000	NA	NA	NA	0.084	0.086	0.019
Bacteria|Bacteroidetes|...|Bacteroidales|[Paraprevotellaceae]	0.394	0.024	0.192	0.061	0.117	0.230	0.165	0.400	0.121	0.001	0.779
Bacteria|Bacteroidetes|...|...|[Paraprevotellaceae]|[Prevotella]	0.397	0.023	0.192	0.061	0.113	0.238	0.165	0.400	0.123	0.001	0.796
Bacteria|Bacteroidetes|...|Bacteroidales|Bacteroidaceae	0.546	0.000	0.072	0.000	0.000	NA	NA	NA	0.157	0.455	0.582
Bacteria|Bacteroidetes|...|...|Bacteroidaceae|Bacteroides	0.551	0.000	0.073	0.000	0.000	NA	NA	NA	0.196	0.559	0.602
Bacteria|Bacteroidetes|...|Bacteroidales|Porphyromonadaceae	0.244	0.104	0.186	0.145	0.096	0.279	0.171	0.419	0.974	0.258	0.099
Bacteria|Bacteroidetes|...|...|Porphyromonadaceae|Porphyromonas	0.319	0.000	0.094	0.003	0.000	NA	NA	NA	0.870	0.264	0.099
Bacteria|Bacteroidetes|...|Bacteroidales|Prevotellaceae	0.355	0.043	0.189	0.080	0.039	0.395	0.148	0.500	0.054	0.024	0.012
Bacteria|Bacteroidetes|...|...|Prevotellaceae|Prevotella	0.355	0.043	0.189	0.080	0.038	0.397	0.148	0.500	0.054	0.024	0.012
Bacteria|Bacteroidetes|Flavobacteriia|Flavobacteriales	0.201	0.165	0.205	0.208	0.196	0.132	0.181	0.400	0.422	0.956	0.049
Bacteria|Bacteroidetes|...|Flavobacteriales|[Weeksellaceae]	0.504	0.000	0.089	0.000	0.000	NA	NA	NA	0.315	0.017	0.088
Bacteria|Bacteroidetes|...|Flavobacteriales|Flavobacteriaceae	0.110	0.305	0.217	0.313	0.222	0.125	0.197	0.400	0.576	0.383	0.102
Bacteria|Bacteroidetes|...|...|Flavobacteriaceae|Capnocytophaga	0.108	0.308	0.217	0.313	0.220	0.127	0.197	0.400	0.579	0.377	0.105
Bacteria|Firmicutes	0.381	0.019	0.170	0.051	0.144	0.155	0.151	0.400	0.190	0.757	0.001

Bacteria|Firmicutes|Bacilli	0.198	0.174	0.205	0.210	0.039	0.402	0.161	0.500	0.026	0.389	0.060
Bacteria|Firmicutes|Bacilli|Gemellales	0.429	0.000	0.086	0.000	0.000	NA	NA	NA	0.292	0.358	0.795
Bacteria|Firmicutes|...|Gemellales|Gemellaceae	0.435	0.000	0.085	0.000	0.000	NA	NA	NA	0.293	0.351	0.791
Bacteria|Firmicutes|...|...|Gemellaceae|Gemella	0.417	0.000	0.086	0.000	0.000	NA	NA	NA	0.228	0.364	0.823
Bacteria|Firmicutes|Bacilli|Lactobacillales	0.182	0.197	0.208	0.222	0.053	0.369	0.163	0.500	0.023	0.408	0.044
Bacteria|Firmicutes|...|Lactobacillales|Carnobacteriaceae	0.250	0.083	0.175	0.134	0.157	0.138	0.153	0.400	0.093	0.014	0.016
Bacteria|Firmicutes|...|...|Carnobacteriaceae|Granulicatella	0.244	0.089	0.175	0.137	0.162	0.130	0.153	0.400	0.095	0.014	0.016
Bacteria|Firmicutes|...|Lactobacillales|Streptococcaceae	0.214	0.162	0.211	0.208	0.004	0.490	0.160	0.500	0.030	0.565	0.064
Bacteria|Firmicutes|...|...|Streptococcaceae|Streptococcus	0.214	0.163	0.211	0.208	0.003	0.492	0.160	0.500	0.030	0.572	0.064
Bacteria|Firmicutes|Clostridia	0.220	0.167	0.223	0.208	0.139	0.209	0.179	0.400	0.522	0.899	0.004
Bacteria|Firmicutes|Clostridia|Clostridiales	0.216	0.171	0.223	0.210	0.141	0.207	0.179	0.400	0.542	0.908	0.005
Bacteria|Firmicutes|...|Clostridiales|[Mogibacteriaceae]	0.247	0.011	0.108	0.038	0.000	NA	NA	NA	0.236	0.037	0.973
Bacteria|Firmicutes|...|Clostridiales|Lachnospiraceae	0.299	0.005	0.116	0.019	0.000	NA	NA	NA	0.130	0.962	0.673
Bacteria|Firmicutes|...|...|Lachnospiraceae|Oribacterium	0.145	0.088	0.110	0.137	0.000	NA	NA	NA	0.007	0.716	0.834
Bacteria|Firmicutes|...|Clostridiales|Peptostreptococcaceae	0.283	0.002	0.101	0.011	0.000	NA	NA	NA	0.916	0.192	0.023
Bacteria|Firmicutes|...|Clostridiales|Veillonellaceae	0.127	0.283	0.219	0.295	0.236	0.078	0.175	0.400	0.448	0.803	0.003
Bacteria|Firmicutes|...|...|Veillonellaceae|Megasphaera	0.164	0.222	0.212	0.246	0.162	0.167	0.176	0.400	0.058	0.309	0.013
Bacteria|Firmicutes|...|...|Veillonellaceae|Selenomonas	0.119	0.250	0.175	0.269	0.106	0.227	0.147	0.400	0.997	0.402	0.620
Bacteria|Firmicutes|...|...|Veillonellaceae|Veillonella	0.162	0.230	0.215	0.252	0.205	0.101	0.171	0.400	0.253	0.644	0.002
Bacteria|Fusobacteria|Fusobacteriia|Fusobacteriales	0.232	0.014	0.107	0.043	0.000	NA	NA	NA	0.169	0.256	0.033
Bacteria|Fusobacteria|...|Fusobacteriales|Fusobacteriaceae	0.147	0.190	0.163	0.221	0.133	0.152	0.135	0.400	0.265	0.883	0.028
Bacteria|Fusobacteria|...|...|Fusobacteriaceae|Fusobacterium	0.150	0.186	0.163	0.220	0.136	0.147	0.136	0.400	0.270	0.890	0.028
Bacteria|Fusobacteria|...|Fusobacteriales|Leptotrichiaceae	0.229	0.126	0.182	0.168	0.029	0.424	0.154	0.500	0.501	0.089	0.224
Bacteria|Fusobacteria|...|...|Leptotrichiaceae|Leptotrichia	0.217	0.122	0.174	0.166	0.086	0.271	0.148	0.419	0.783	0.092	0.278
Bacteria|Proteobacteria	0.347	0.039	0.185	0.079	0.110	0.248	0.167	0.401	0.140	0.048	0.004
Bacteria|Proteobacteria|Betaproteobacteria	0.325	0.041	0.178	0.080	0.179	0.125	0.165	0.400	0.610	0.665	0.017
Bacteria|Proteobacteria|Betaproteobacteria|Burkholderiales	0.080	0.197	0.096	0.222	0.000	NA	NA	NA	0.077	0.439	0.032
Bacteria|Proteobacteria|...|Burkholderiales|Burkholderiaceae	0.054	0.269	0.090	0.285	0.000	NA	NA	NA	0.097	0.666	0.034

Bacteria|Proteobacteria|Betaproteobacteria|Neisseriales|Neisseriaceae	0.332	0.037	0.177	0.077	0.173	0.133	0.164	0.400	0.614	0.633	0.019
Bacteria|Proteobacteria|...|...|Neisseriaceae|Eikenella	0.037	0.430	0.212	0.430	0.258	0.069	0.180	0.400	0.513	0.978	0.146
Bacteria|Proteobacteria|...|...|Neisseriaceae|Neisseria	0.308	0.047	0.177	0.086	0.179	0.125	0.164	0.400	0.728	0.574	0.022
Bacteria|Proteobacteria|Epsilonproteobacteria	0.204	0.104	0.154	0.145	0.014	0.463	0.149	0.500	0.172	0.396	0.478
Bacteria|Proteobacteria|Epsilonproteobacteria|Campylobacterales	0.204	0.104	0.154	0.145	0.014	0.463	0.149	0.500	0.171	0.398	0.476
Bacteria|Proteobacteria|...|Campylobacterales|Campylobacteraceae	0.206	0.100	0.153	0.145	0.014	0.462	0.149	0.500	0.173	0.400	0.470
Bacteria|Proteobacteria|...|...|Campylobacteraceae|Campylobacter	0.204	0.102	0.153	0.145	0.013	0.464	0.148	0.500	0.179	0.390	0.456
Bacteria|Proteobacteria|Gammaproteobacteria	0.200	0.026	0.104	0.061	0.000	NA	NA	NA	0.094	0.002	0.024
Bacteria|Proteobacteria|Gammaproteobacteria|Enterobacteriales|Enterobacteriaceae	0.367	0.000	0.091	0.000	0.000	NA	NA	NA	0.016	0.807	0.968
Bacteria|Proteobacteria|...|Enterobacteriales|Enterobacteriaceae|Providencia	0.698	0.000	0.058	0.000	0.000	NA	NA	NA	0.296	0.995	0.397
Bacteria|Proteobacteria|Gammaproteobacteria|Pasteurellales|Pasteurellaceae	0.173	0.051	0.108	0.090	0.000	NA	NA	NA	0.226	0.003	0.036
Bacteria|Proteobacteria|...|...|Pasteurellaceae|Actinobacillus	0.222	0.008	0.095	0.030	0.000	NA	NA	NA	0.113	0.011	0.338
Bacteria|Proteobacteria|...|...|Pasteurellaceae|Aggregatibacter	0.253	0.003	0.094	0.013	0.000	NA	NA	NA	0.647	0.970	0.287
Bacteria|Proteobacteria|...|...|Pasteurellaceae|Haemophilus	0.209	0.026	0.111	0.061	0.000	NA	NA	NA	0.337	0.004	0.049
Bacteria|Spirochaetes	0.231	0.009	0.102	0.035	0.000	NA	NA	NA	0.686	0.461	0.487
Bacteria|Spirochaetes|Spirochaetes	0.231	0.080	0.158	0.132	0.000	0.500	0.122	0.500	0.683	0.470	0.485
Bacteria|Spirochaetes|Spirochaetes|Spirochaetales|Spirochaetaceae	0.231	0.080	0.158	0.132	0.000	0.500	0.122	0.500	0.683	0.470	0.485
Bacteria|Spirochaetes|...|...|Spirochaetaceae|Treponema	0.231	0.080	0.158	0.132	0.000	0.500	0.122	0.500	0.683	0.470	0.485

H2r: total additive genetic heritability; p.H2r: significance of H2r; se.H2r: H2r standard error; FDR.H2r: FDR-corrected H2r p-value; C2: common/shared environment; p.C2: significance of C2; se.C2: C2 standard error; FDR.C2: FDR-corrected C2 p-value; p.sex, p.age, p.PY: significance of each covariate.

Supplementary Table S2. Complete list of significant associations of lifestyle factors with microbial abundances.

Variable	Feature	Coefficient	p-value	q-value
Alcohol consumption (g/week)	Bacteria|Actinobacteria|Actinobacteria|Actinomycetales|Actinomycetaceae	0.00006	0.00894	0.12184
Alcohol consumption (g/week)	Bacteria|Actinobacteria|Actinobacteria|Actinomycetales|Actinomycetaceae|Actinomyces	0.00006	0.00945	0.12184
Physical activity (MET·min/week)	Bacteria|Firmicutes|Clostridia|Clostridiales|Lachnospiraceae	0.00000	0.01088	0.12875
Smoking (Pack-years)	Bacteria|Bacteroidetes|Bacteroidia|Bacteroidales|Prevotellaceae	0.00383	0.01781	0.16640
Smoking (Pack-years)	Bacteria|Bacteroidetes|Bacteroidia|Bacteroidales|Prevotellaceae|Prevotella	0.00383	0.01780	0.16640
Smoking (Pack-years)	Bacteria|Bacteroidetes|Flavobacteriia|Flavobacteriales	-0.00119	0.01540	0.15618
Smoking (Pack-years)	Bacteria|Firmicutes	0.00261	0.01228	0.13626
Smoking (Pack-years)	Bacteria|Firmicutes|Clostridia	0.00381	0.00077	0.03402
Smoking (Pack-years)	Bacteria|Firmicutes|Clostridia|Clostridiales	0.00381	0.00077	0.03402
Smoking (Pack-years)	Bacteria|Firmicutes|Clostridia|Clostridiales|Peptostreptococcaceae	-0.00027	0.02319	0.21109
Smoking (Pack-years)	Bacteria|Firmicutes|Clostridia|Clostridiales|Veillonellaceae	0.00394	0.00061	0.03402
Smoking (Pack-years)	Bacteria|Firmicutes|Clostridia|Clostridiales|Veillonellaceae|Megasphaera	0.00154	0.00115	0.03653
Smoking (Pack-years)	Bacteria|Firmicutes|Clostridia|Clostridiales|Veillonellaceae|Veillonella	0.00346	0.00123	0.03653
Smoking (Pack-years)	Bacteria|Proteobacteria	-0.00463	0.00831	0.12184
Smoking (Pack-years)	Bacteria|Proteobacteria|Betaproteobacteria|Burkholderiales	-0.00040	0.01538	0.15618
Smoking (Pack-years)	Bacteria|Proteobacteria|Betaproteobacteria|Burkholderiales|Burkholderiaceae	-0.00037	0.02391	0.21219
Smoking (Pack-years)	Bacteria|Proteobacteria|Betaproteobacteria|Neisseriales|Neisseriaceae|Eikenella	-0.00068	0.00451	0.09418
Smoking (Pack-years)	Bacteria|Proteobacteria|Gammaproteobacteria	-0.00409	0.00109	0.03653
Smoking (Pack-years)	Bacteria|Proteobacteria|Gammaproteobacteria|Pasteurellales|Pasteurellaceae	-0.00375	0.00199	0.04701
Smoking (Pack-years)	Bacteria|Proteobacteria|Gammaproteobacteria|Pasteurellales|Pasteurellaceae|Haemophilus	-0.00297	0.00566	0.11169

Supplementary Table S3. Significant associations of smoking status with microbial abundances.

Variable	Feature	Value	Coefficient	p-value	q-value
Smoking status	Bacteria|Actinobacteria	Ex-smoker	-0.03885	0.02075	0.21477
Smoking status	Bacteria|Actinobacteria|Actinobacteria	Ex-smoker	-0.04004	0.01771	0.19895
Smoking status	Bacteria|Actinobacteria|Actinobacteria|Actinomycetales	Ex-smoker	-0.03975	0.01827	0.19907
Smoking status	Bacteria|Actinobacteria|Actinobacteria|Actinomycetales|Micrococcaceae	Ex-smoker	-0.04864	0.00196	0.04681
Smoking status	Bacteria|Actinobacteria|Actinobacteria|Actinomycetales|Micrococcaceae|Rothia	Ex-smoker	-0.04863	0.00196	0.04681
Smoking status	Bacteria|Actinobacteria|Coriobacteriia|Coriobacteriales|Coriobacteriaceae	Current-smoker	0.01088	0.00228	0.04681
Smoking status	Bacteria|Actinobacteria|Coriobacteriia|Coriobacteriales|Coriobacteriaceae|Atopobium	Current-smoker	0.01092	0.00217	0.04681
Smoking status	Bacteria|Firmicutes	Current-smoker	0.06393	0.00122	0.03875
Smoking status	Bacteria|Firmicutes|Clostridia	Current-smoker	0.07468	0.00048	0.02223
Smoking status	Bacteria|Firmicutes|Clostridia|Clostridiales	Current-smoker	0.07469	0.00048	0.02223
Smoking status	Bacteria|Firmicutes|Clostridia|Clostridiales|Veillonellaceae	Current-smoker	0.07763	0.00035	0.02068
Smoking status	Bacteria|Firmicutes|Clostridia|Clostridiales|Veillonellaceae|Megasphaera	Current-smoker	0.03706	0.00002	0.00776
Smoking status	Bacteria|Firmicutes|Clostridia|Clostridiales|Veillonellaceae|Veillonella	Current-smoker	0.06379	0.00182	0.04681
Smoking status	Bacteria|Proteobacteria	Current-smoker	-0.09477	0.00537	0.08898
Smoking status	Bacteria|Proteobacteria|Betaproteobacteria	Current-smoker	-0.07647	0.02263	0.22715
Smoking status	Bacteria|Proteobacteria|Betaproteobacteria|Burkholderiales	Current-smoker	-0.01030	0.00067	0.02761
Smoking status	Bacteria|Proteobacteria|Betaproteobacteria|Burkholderiales|Burkholderiaceae	Current-smoker	-0.00969	0.00099	0.03416
Smoking status	Bacteria|Proteobacteria|Betaproteobacteria|Neisseriales|Neisseriaceae	Current-smoker	-0.07487	0.02603	0.24962

Supplementary Table S4. Significant associations of Fagerstrom Test of Nicotine Dependence (FTND) scores with microbial abundances.

Variable	Feature	Coefficient	p-value	q-value
Smoking (FTND)	Bacteria|Firmicutes|Clostridia	0.01232	0.01464	0.17201
Smoking (FTND)	Bacteria|Firmicutes|Clostridia|Clostridiales	0.01232	0.01465	0.17201
Smoking (FTND)	Bacteria|Firmicutes|Clostridia|Clostridiales|Veillonellaceae	0.01293	0.01172	0.17201
Smoking (FTND)	Bacteria|Firmicutes|Clostridia|Clostridiales|Veillonellaceae|Megasphaera	0.00663	0.00148	0.08767
Smoking (FTND)	Bacteria|Firmicutes|Clostridia|Clostridiales|Veillonellaceae|Veillonella	0.01068	0.02511	0.21743
Smoking (FTND)	Bacteria|Proteobacteria	-0.01717	0.02845	0.23488
Smoking (FTND)	Bacteria|Proteobacteria|Gammaproteobacteria	-0.01434	0.00911	0.17201
Smoking (FTND)	Bacteria|Proteobacteria|Gammaproteobacteria|Pasteurellales|Pasteurellaceae	-0.01327	0.01269	0.17201
Smoking (FTND)	Bacteria|Proteobacteria|Gammaproteobacteria|Pasteurellales|Pasteurellaceae|Haemophilus	-0.01209	0.01026	0.17201

FTND values were considered a continuous variable.
